# Editorial: Synergistic interaction of plants and microbes for removal of toxic elements/chemicals: multidisciplinary approaches for a sustainable environment

**DOI:** 10.3389/fmicb.2023.1222164

**Published:** 2023-06-30

**Authors:** Pooja Sharma, Surendra Pratap Singh, Hafiz M. N. Iqbal, Yen Wah Tong

**Affiliations:** ^1^NUS Environmental Research Institute, National University of Singapore, Singapore, Singapore; ^2^Energy and Environmental Sustainability for Megacities (E2S2) Phase II, Campus for Research Excellence and Technological Enterprise (CREATE), Singapore, Singapore; ^3^Plant Molecular Biology Laboratory, Department of Botany, Dayanand Anglo-Vedic (PG) College, Chhatrapati Shahu Ji Maharaj University, Kanpur, India; ^4^Tecnologico de Monterrey, School of Engineering and Sciences, Monterrey, Mexico; ^5^Tecnologico de Monterrey, Institute of Advanced Materials for Sustainable Manufacturing, Monterrey, Mexico; ^6^Department of Chemical and Biomolecular Engineering, National University of Singapore, Singapore, Singapore

**Keywords:** microbes, toxic elements/chemicals, sustainable environment, removal, bioremediation (soil and groundwater)

## Summary

Toxic elements and chemicals (TECh) in the environment can have ecological effects (disrupting ecosystems, affecting biodiversity) and potential health effects (cancer, neurological and immune system disorders, and other chronic diseases). They can be released into the environment by natural processes or through human activities, such as industrial processes, agriculture, mining, and transportation. TECh include heavy metals, persistent organic pollutants, pesticides, industrial chemicals, and radioactive materials. As part of the bioremediation process, microorganisms play an important role in removing TECh from the environment through metabolic transformation, adsorption, and volatilization. Microbes can convert TECh into less harmful compounds for energy, while others can bind to them and reduce their toxicity. A wide range of environmental applications uses bioremediation, including cleaning contaminated soil, groundwater, and surface water. Compared to traditional environmental remediation methods such as excavation and incineration, it is considered more sustainable and cost-effective. In addition to the type and concentration of contaminants, the properties of the soil or water, and the availability of nutrients and oxygen can all affect the effectiveness of bioremediation. The synergistic interactions between plants and microbes can enhance the removal of TECh from the environment (Segura and Ramos, 2013). By releasing compounds from their roots, plants can attract beneficial microbes to their root zones, and these microbes can form mutualistic relationships with the plants, enhancing their growth and survival and degrading or immobilizing toxic substances in the soil. In addition to assisting phytoremediation directly, microbes can also be added as inoculants to contaminated soil or water to enhance the phytoremediation process by breaking down toxic substances into less harmful forms or immobilizing them via adsorption or precipitation (Ratna et al., 2021; Sharma, 2021).

The composition of the rhizosphere bacterial community and the response of soil physiochemical properties to nitrogen applications can provide valuable information about the ecology and function of the soil microbial community and improve soil health and fertility (Khan et al.). Based on whole-genome and transcriptome sequencing, Kashif et al. report that whole-genome and transcriptome sequencing can provide valuable insight into the metabolic potential and functional diversity of microbial communities. A study conducted by Muhammad et al. indicates that soil microbial communities can reduce greenhouse gas emissions while improving soil health and sequestering carbon. However, as discussed by Jalmi and Sinha, there remains uncertainty as to the effects of plant growth-promoting rhizobacteria (PGPR) on plant signaling and stress management. The study by Zhao et al. reports on soil carbon sequestration and bacterial community in calcareous soils by using straw and wood ash. Another study by Ali et al. examines the effect of biochar and manure applications on class 1 integrons, antimicrobial resistance, and gene cassette diversity in paddy soils. Paddy soil's abundance and diversity of integrons (genetic elements that can capture and mobilize antimicrobial resistance genes), antimicrobial resistance, and gene cassettes (the genetic units that encode resistance genes) are affected by biochar and manure addition. in addition to improving soil health and crop productivity, Song et al. report on the potential benefits and drawbacks of biochar and organic-inorganic fertilizers in agricultural settings. Pereira et al. consider the implications of developing sustainable and environmentally friendly approaches for removing heavy metals from contaminated water sources and producing valuable chemicals using microbial biocatalysts.

This Research Topic brings together eight papers, including research and review articles submitted by authors from around the world. Their contribution to high-quality work in microbiology is greatly appreciated by the scientific community and will help in managing toxic chemicals through microbes for environmental clean-up. We also thank all the reviewers for their excellent work. Lastly, we would like to thank the Editors-in-Chief, Research Topics Editors, and Journal Managers at Frontiers for their encouragement and support during this process.

## Author contributions

PS, SS, and HI: conceptualization, writing—original draft, and editing and reviewing. YT: conceptualization, writing—original draft, funding acquisition, project administration, and editing and reviewing. All authors contributed to the article and approved the submitted version.

## References

[B1] Ratna S. Rastogi S. Kumar R. (2021). “Phytoremediation: a synergistic interaction between plants and microbes for removal of unwanted chemicals/contaminants,” in Microbes and Signaling Biomolecules Against Plant Stress: Strategies of Plant-Microbe Relationships for Better Survival, ed A. Sharma (Singapore: Springer Singapore), 199–222. 10.1007/978-981-15-7094-0_11

[B2] Segura A. Ramos J. L. (2013). Plant–bacteria interactions in the removal of pollutants. Curr. Opin. Biotechnol. 24, 467–473. 10.1016/j.copbio.2012.09.011 23098915

[B3] Sharma P. (2021). Efficiency of bacteria and bacterial assisted phytoremediation of heavy metals: an update. Bioresour. Technol. 328, 124835. 10.1016/j.biortech.2021.124835 33618184

